# Reply to Farahani, P.; Wahid, L. Comment on “Viderman et al. Remote Monitoring of Chronic Critically Ill Patients after Hospital Discharge: A Systematic Review. *J. Clin. Med.* 2022, *11*, 1010”

**DOI:** 10.3390/jcm12216797

**Published:** 2023-10-27

**Authors:** Dmitriy Viderman, Elena Seri, Mina Aubakirova, Yerkin Abdildin, Rafael Badenes, Federico Bilotta

**Affiliations:** 1 Department of Surgery, Section of Anesthesiology, Intensive Care, and Pain Medicine, Nazarbayev University School of Medicine, Astana 010000, Kazakhstan; dmitriy.viderman@nu.edu.kz; 2 Department of Anesthesiology, Critical Care and Pain Medicine, Policlinico Umberto I, “Sapienza” University of Rome, 00185 Rome, Italy; eliseri2499@gmail.com (E.S.); bilotta@tiscali.it (F.B.); 3 Department of Biomedical Sciences, Nazarbayev University School of Medicine, Astana 010000, Kazakhstan; mina.aubakirova@nu.edu.kz; 4 Department of Mechanical and Aerospace Engineering, School of Engineering and Digital Sciences, Nazarbayev University, Astana 010000, Kazakhstan; yerkin.abdildin@nu.edu.kz; 5 Department of Anesthesiology and Surgical-Trauma Intensive Care, Hospital Clínic Universitari, University of Valencia, 46010 Valencia, Spain

Thank you very much for taking the time to read this systematic review and for sharing your thoughts. We very much appreciate your interest. You raised several important points [1].

The initial objective of our systematic review was to explore the evidence on the potential application of remote monitoring in chronic critically ill patients with brain injuries after hospital discharge [2]. However, since there was a high heterogeneity of the patient population and the original studies included not only critically ill patients with brain injuries but also patients without brain injuries, as well as patients with a combination of several cardiovascular, respiratory, and neurological conditions, and in some studies the patient population was not properly described or specified, there was not enough evidence to support our initial objective. Therefore, we had to slightly broaden our objective to critically ill patients in general.

We pointed out several important limitations of this systematic review, including the high heterogeneity of the original studies. Many of the included studies were performed by researchers with different backgrounds, such as engineering, computer science, as well as medicine; therefore, the objectives of these studies had different focuses, interests, methodologies, and subsequently results. Most of the included studies mainly focused on technical rather than clinical characteristics. Therefore, instead of making strong conclusions and recommendations, we primarily reported general issues and potential solutions. Also, due to the high level of heterogeneity in the study objectives and methods, we did not conduct a meta-analysis.

These limitations can be addressed through closer collaboration between clinicians, investigators, caregivers, computer scientists, engineers, and information technology professionals. Such a collaboration can reduce the existing gaps in knowledge. Also, future clinical trials in this area are warranted before such technologies are widely implemented into clinical practice. As previously suggested, these clinical trials should be conducted using a multidisciplinary approach. 

We believe that this review provided readers with some important information, including the general basics regarding using remote monitoring, monitoring of physiological parameters, glucose monitoring, and neurological monitoring, which might be used in such patients after their discharge from the hospital, although more data are needed.

We also highlighted the existing issues associated with using remote monitoring technologies, such as patients reporting that these devices were “uncomfortable to wear”, “difficult to wear while performing activities”, “difficult to wear while eating”, “difficult to wear while washing hands”; patients also reported “anxiety over possible injury and pressure sores”, while some patients were “concerned that these devices would replace face-to-face interaction with nurses” [3,4,5,6,7].

We also want to point out that the quality and functionality of these devices might have improved significantly since the original studies were conducted and published.

The available data show that wearable devices might be used to monitor blood pressure, pulse, heart rate, respiratory rate, oxygen saturation, and some metabolic parameters. 

Certainly, more original studies are required to support the initial objective of this systematic review. We agree that further systematic reviews with specific inclusion and exclusion criteria are required to address such health issues after more evidence is generated. We are open to any suggestions and truly appreciate your input on potential future directions. Should you have further comments, questions, or ideas to discuss, we welcome the opportunity to engage in further dialogue.

Your comments and feedback contribute to the conversation in this field, and we look forward to the exchange of ideas.

Many thanks for your engagement and attentive comments.
